# Protocol for a systematic review of reporting standards of lower limb endovascular interventions in peripheral arterial disease

**DOI:** 10.1186/s13643-023-02182-9

**Published:** 2023-02-15

**Authors:** Ewa M. Zywicka, Lucy Elliott, Christopher P. Twine, Ronelle Mouton, Robert J. Hinchliffe

**Affiliations:** 1 Translational Health Sciences, University of Bristol Medical School, Bristol, UK; 2 Southmead Hospital, North Bristol NHS Trust, Bristol, UK; 3 Centre for Surgical Research, University of Bristol Medical School, Bristol, UK

**Keywords:** Endovascular intervention, Angioplasty, Peripheral arterial disease, Reporting standards

## Abstract

**Introduction:**

Techniques and devices for the endovascular treatment of peripheral arterial disease (PAD) are continuously evolving. High-quality clinical trials limit the variation in how endovascular interventions are described, performed and reported.

The aim of this systematic review is to assess the quality of reporting standards in randomised controlled trials (RCTs) of endovascular lower limb interventions against the Consolidated Standards of Reporting Trials for Non-Pharmacologic Treatments (CONSORT-NPT) and template for intervention description and replication (TIDieR) framework.

**Methods:**

Randomised trials including participants with peripheral arterial disease undergoing any infra-inguinal lower limb endovascular arterial intervention, searched from Medline, Embase and Cochrane Library databases from inception to December 2021, will be included. All study data, including details of the procedure investigated, will be extracted in keeping with the CONSORT-NPT and TIDieR checklist. Descriptive statistics will be used to summarise general study details and reporting standards of the trials.

**Discussion:**

The results will be used to inform the design of future RCTs in this area by optimising the way the interventions are described, standardised, and monitored. The systematic review will be disseminated via peer-reviewed manuscripts and presentations at relevant conferences.

**Systematic review registration:**

PROSPERO CRD42022288214

**Supplementary Information:**

The online version contains supplementary material available at 10.1186/s13643-023-02182-9.

## Background

Endovascular treatment of peripheral arterial disease (PAD) is a rapidly evolving field with new innovative techniques and devices being introduced to clinical practice on a regular basis. Well-designed randomised controlled trials (RCTs) are widely accepted as the gold standard in evaluating endovascular interventions before being adopted in clinical practice. Still, some unresolved issues in trial design, standardisation and reporting may affect the quality of these studies [1, 2]. An unclear description of the intervention being investigated, for example, prevents understanding what exact procedure was investigated within the clinical trial, comparing results between different interventions, and implementing successful procedures in clinical practice [1].

The issue of incomplete and inadequate reporting in interventional (and non-interventional) trials is a well-recognised problem and was challenged by the introduction of the Consolidated Standards of Reporting Trials for Non-Pharmacologic Treatments (CONSORT-NPT) and its specific extension for the description of complex interventions with the TIDieR checklists [3–6].

A recent analysis of the description of complex interventions in surgery and anaesthesia RCTs demonstrated poor reporting standards and poor adherence to CONSORT-NPT guidance. These studies suggested that the current guidance might not be adequate for RCTs involving complex interventions and that more comprehensive and specific frameworks for intervention descriptions should be developed [7, 8].

The quality of clinical trials evaluating endovascular interventions has not been specifically investigated, and it is not known if the CONSORT-NPT and TIDieR guidance are being adopted [3–6]. A careful review of reporting standards in RCTs investigating endovascular lower limb interventions could identify potential areas for improvement in endovascular research.

### Study aim

The aim of this systematic review is to assess the quality of reporting standards in infra-inguinal endovascular lower limb interventions for the treatment of atherosclerotic PAD in randomised controlled trials (RCTs) against the CONSORT-NPT and TIDieR checklist.

## Methods

This systematic review protocol follows the Preferred Reporting Items for Systematic Review and Meta-Analysis Protocols (PRISMA-P) guidelines [9]. It was registered with the International Prospective Register of Systematic Reviews (PROSPERO: CRD42022288214) and will be updated with amendments if required.

### Data sources and search strategy

MEDLINE, EMBASE and the Cochrane library databases will be searched via Ovid from inception to December 2021 to identify randomised trials including patients undergoing any infra-inguinal endovascular intervention for atherosclerotic chronic peripheral arterial disease. Trial registry databases including ClinicalTrials.gov, ICTRP, and ISRCTN will be searched separately. There will be no limit on the country of study but only studies written in English will be included. No searching restriction on date or publication status will be applied. A draft of the search strategy is provided as Additional file 1.

### Study selection inclusion and exclusion criteria

Randomised controlled trials including human participants with chronic atherosclerotic PAD of the lower limb undergoing any endovascular intervention as main therapy or as adjunctive therapy will be included. Studies, where endovascular interventions are being evaluated in cadavers, laboratories and animals, will be excluded.

Trials studying patients with PAD treated by open surgery or non-interventional treatments will be excluded. Trials including patients with non-atherosclerotic lower limb disease such as aneurysms will be excluded. Trials focusing on the treatment of patients with acute limb ischemia will also be excluded.

Duplicate records will be excluded, and the PRISMA flowchart will be used to present the selection process.

### Study management

Electronic article information will be downloaded into Rayyan Software (Qatar Computing Research Institute, HBKU, Doha, Qatar) [10]. Study selection will be performed by screening titles and abstracts. After title and abstract screening, the full-text articles will be screened against inclusion criteria by two researchers independently. Disagreements will be resolved through discussion with the senior researcher/other members of the research team.

The authors of included studies will be contacted to obtain additional study protocol if available.

### Data extraction and assessment

Data will be extracted using a prespecified, standardised extraction form. The data to be extracted will be based on the CONSORT-NPT and the TIDiER checklists, including general study information, intervention description, standardisation and adherence, and expertise of care providers [3–6]. Data will be collected and managed using the REDCap electronic data capture tool hosted at the University of Bristol, UK [11, 12]. Data extraction will be completed by one researcher and verified by a second independent researcher. Disagreements will be resolved through discussion with the senior researcher/other members of the research team.

#### General study information (CONSORT-NPT items 3, 4, 6, 23 and 25)

Descriptions of the following general study details will be recorded: journal and year of publication, trial design, availability of study protocol and its format, number of included patients together with inclusion and exclusion criteria, Rutherford classification of disease severity, primary and secondary endpoints, the type of endovascular intervention investigated, and the target artery being treated [13].

#### Intervention description (CONSORT-NPT items 5 and 5a, TIDieR items 4, 6, 8, 9 and 10)

The endovascular interventions described in each study will be recorded in keeping with the CONSORT-NPT and TIDieR checklists in as much detail as is published in either the included study or extracted study protocol.

The intervention description will be recorded according to an initial draft of the typology of endovascular interventions developed by the authors. The intervention description will be divided into pre-procedure interventions, procedure components (arterial access, crossing lesion, treating lesion, and closure of artery) and post-procedure interventions, including pharmacological co-interventions administered (antiplatelet/anticoagulation/anaesthesia/analgesia/vasodilators).

All studies providing information about any aspect of the intervention will be classified as providing a description, regardless of the level of detail. Further classification will be recorded about the level of details provided (none/some/precise). The presence of citations to other materials describing the intervention in more detail will be recorded separately.

If the study allows deviations, personalisation/tailoring of the procedure to participants, additional interventions or bailout procedures, details of variables used for participant assessment and description of tailoring options will be recorded. Assessors will record judgements about whether enough information is provided to be able to replicate device use in routine practice (yes/no/unsure).

#### Intervention standardisation and adherence (CONSORT-NPT item 5b, 5c and 5d, TIDieR items 11 and 12)

Attempts to standardise the endovascular intervention or comparator will be recorded including details about why and how this was done and according to which standard. Standardisation will be defined as a process “to establish a standard consisting of regulations for how something is to be done” [14].

Attempts to assess or enhance adherence of care providers or participants to study protocol will also be recorded. Reporting of fidelity to study protocol will be recorded for each study including details of any strategies that were used to improve fidelity and details of how this was measured. Fidelity will be defined as “how far those responsible for delivering an intervention actually adhere to the intervention as it is outlined by its designers” as described by Blencowe et al. [7].

#### Clinician expertise (CONSORT-NPT items 3 and 15, TIDieR item 5)

Details regarding location and type of centres providing intervention (primary, secondary, tertiary), experience of care providers (case volume, qualification, years of experience or level of training), any eligibility criteria for centres or care providers and any specific training provided will be recorded.

### Assessment of risk of bias

The Cochrane Collaboration’s risk of bias tool will be used to evaluate bias in all selected RCTs by two researchers [15]. This includes an assessment of sequence generation and allocation concealment, blinding of participants and outcome assessment, incomplete outcome data and selective outcome reporting.

### Data analysis

A PRISMA flow chart of search and study selection with included and excluded studies will be presented and reasons for exclusion will be given.

Percentage statistics will be used to summarise reporting standards of the included trials. A narrative synthesis will summarise the results by organising the data into relevant extraction themes.

Formal statistical comparison will not be undertaken because the overall aim of this review is to summarise reporting standards and not to analyse specific trial results. The same approach has been taken in other published systematic reviews that have analysed reporting standards in other research fields [7, 8].

### Patient and public involvement

There was no involvement of patients or the public in the design of this systematic review and no patient or public members will be required to complete the review.

It is important to note that recently The Vascular James Lind Alliance Priority Setting Partnership, including both patients and health care professionals, agreed on a priority list for PAD research and priority number one was the answer to the question “What can be done to improve outcomes in patients with severe circulation problems to their legs?” [16]. We believe that improvement of the quality of RCTs about the treatment of patients with PAD will have a significant impact on the improvement of patient outcomes. 

## Supplementary Information

**Additional file 1.** Complete search strategy.

## Data Availability

Not applicable.
