# Supplementary material for: Protocol for a systematic review of reporting standards of lower limb endovascular interventions in peripheral arterial disease
Source: Syst Rev. 2023 Feb 15;12:20. doi: 10.1186/s13643-023-02182-9 (PMC9930275; doi:10.1186/s13643-023-02182-9)
Supplement: Supplementary file 1 — Additional file 1. Complete search strategy. [file 13643_2023_2182_MOESM1_ESM.docx]

**Additional file 1 : Complete search strategy**

**MEDLINE (Ovid) search strategy**

| ID | Search |
| --- | --- |
| #1 | randomized controlled trial.pt. |
| #2 | (RCT or randomi#ed or randomi#ation).ab,ti,kf. |
| #3 | (random* adj3 (administ* or allocat* or assign* or class* or control* or determine* or divide* or distribut* or expose* or fashion* or number* or place* or recruit* or subsitut* or treat*)).ti,ab,kf. |
| #4 | trial.ti,kf. |
| #5 | ((singl* or doubl* or tripl* or trebl*) adj3 (blind* or dummy)).mp. |
| #6 | clinical trial, phase ii/ or clinical trial, phase iii/ or clinical trial, phase iv/ or randomized controlled trial/ or pragmatic clinical trial/ |
| #7 | placebo.mp. and surgery.fs. |
| #8 | (control* adj3 (trial or study or group?)).ti,ab,tw. |
| #9 | randomized controlled trial/ |
| #10 | clinical trial.hw. and (sham or placebo or random* or control* or group?).mp. |
| #11 | or/1-10 |
| #12 | exp Peripheral Vascular Disease/ |
| #13 | *Arterial Occlusive Diseases/ or Arterial Occlusive Diseases/pa, su, th |
| #14 | Arteriosclerosis Obliterans/pa, su, th |
| #15 | (Atherosclerosis/pa, su, th or Arteriosclerosis/pa, su, th) and (peripher* or limb? or leg?).ti,ab,kf,hw. |
| #16 | *Femoral Artery/ or Femoral Artery/pa, su, th |
| #17 | *Popliteal Artery/ or Popliteal Artery/pa, su, th |
| #18 | *Iliac Artery/ or Iliac Artery/pa, su, th |
| #19 | *Tibial Arteries/ or Tibial Arteries/pa, su, th |
| #20 | (Femoral Artery/ or Popliteal Artery/ or Iliac Artery/ or Tibial Arteries/) and (Vascular Patency/ or Vascular Surgical Procedures/) |
| #21 | (Limb Salvage/ or Leg/bs or Foot/bs or Lower Extremity/bs) and (Ischemia/ or Revascularization/ or Thrombosis/) |
| #22 | ((limb? or leg? or foot or feet or lower extremit* or ((above or below) adj2 (knee? or ankle?))) adj3 (arter* or isch?emi* or lesion? or recanali* or revascular* or segment or thrombo* or surgery or intervention)).ti,ab,kf. |
| #23 | (peripheral adj3 (arter* or vascular*) adj3 (disease or isch?emi* or segment or occlus* or patency or reocclus* or re-occlus* or steno* or restenos* or revascular* or recanali* or obstruct* or lesio* or block* or harden* or stiffen* or obliter* or intervention or surgery)).ti,ab,kf. |
| #24 | (((femoral or femoropopliteal or femor* popliteal or infrapopliteal or infra popliteal or iliac or aortoiliac or aorto iliac or tibial or tibioperoneal or tibio peroneal or tibia fibila? or tibiafibila? or crural or inguinal or inguinal or infrainguinal or pedis or tarsal or planar or malleolar or arcuate or metatarsal) adj2 (arter* or segment)) and (disease or isch?emi* or occlus* or patency or reocclus* or re-occlus* or steno* or restenos* or revascular* or recanali* or obstruct* or lesio* or block* or harden* or stiffen* or obliter* or intervention or surgery)).ti,ab,kf. |
| #25 | ((femoral or femoropopliteal or femor* popliteal or infrapopliteal or infra popliteal or iliac or aortoiliac or aorto iliac or tibial or tibioperoneal or tibio peroneal or tibia fibila? or tibiafibila? or crural or inguinal or inguinal or infrainguinal or pedis or tarsal or planar or malleolar or arcuate or metatarsal) adj3 (isch?emi* or occlus* or patency or reocclus* or re-occlus* or steno* or restenos* or revascular* or recanali* or obstruct* or lesion* or block* or harden* or stiffen* or obliter* or intervention or surgery)).ti,ab,kf. |
| #26 | ((critical or chronic) adj2 limb? adj2 isch?emi*).ti,ab,kf,hw. |
| #27 | claudication.ti,ab,kf,hw. |
| #28 | or/12-27 |
| #29 | ((femoral or femoropopliteal or femor* popliteal or infrapopliteal or infra popliteal or iliac or aortoiliac or aorto iliac or tibial or tibioperoneal or tibio peroneal or tibia fibila? or tibiafibila? or crural or inguinal or inguinal or infrainguinal or pedis or tarsal or planar or malleolar or arcuate or metatarsal or ((above or below) adj2 (knee? or ankle?))) adj3 (balloon? or baloon? or angioplas* or stent*)).ti,ab,kf. |
| #30 | ((Admiral or Amphirion or Apex or Chocolate or Cotavance or CVI or Dior or Elutax or EverCross or Freeway or Genie or "IN.PACT" or LegFlow or Lutonix or Moxy or Pacific or Pantera or Range or SeQuent or Stellarex or Zilver) adj5 (balloon? or baloon? or catheter* or stent*)).ti,ab,kf. |
| #31 | ((Admiral or Amphirion or Apex or Chocolate or Cotavance or CVI or Dior or Elutax or EverCross or Freeway or Genie or "IN.PACT" or LegFlow or Lutonix or Moxy or Pacific or Pantera or Range or SeQuent or Stellarex or Zilver) adj5 (femoral or femoropopliteal or femor* popliteal or infrapopliteal or infra popliteal or iliac or aortoiliac or aorto iliac or tibial or tibioperoneal or tibio peroneal or tibia fibila? or tibiafibila? or crural or inguinal or inguinal or infrainguinal or pedis or tarsal or planar or malleolar or arcuate or metatarsal or ((above or below) adj2 (knee? or ankle?)))).ti,ab,kf. |
| #32 | ((peripheral or percutaneous) adj3 endovascular).ti,ab,kf. |
| #33 | or/29-32 |
| #34 | angioplasty/ or angioplasty, balloon/ or angioplasty, balloon, coronary/ or angioplasty, balloon, laser-assisted/ or angioplasty, laser/ |
| #35 | (angioplas* or venoplas* or (percutaneous adj3 (intervention? or procedure? or technique? or treatment?))).ti,ab,kf. |
| #36 | (balloon? or baloon? or dilatation catheter*).ti,ab,kf. |
| #37 | (PTA or DEB or DCB or PEB or PCB).ti,ab,kf. and (femoral or femoropopliteal or femor* popliteal or infrapopliteal or infra popliteal or iliac or aortoiliac or aorto iliac or tibial or tibioperoneal or tibio peroneal or tibia fibila? or tibiafibila? or crural or inguinal or inguinal or infrainguinal or pedis or tarsal or planar or malleolar or arcuate or metatarsal or ((above or below) adj2 (knee? or ankle?))).ti,ab,kf,hw. |
| #38 | (paclitaxel or PTX).ti,ab,kf,hw. |
| #39 | Endovascular Procedures/ |
| #40 | endovascular.ti. or (endovascular* adj (intervention? or procedure? or technique? or treatment?)).ti,ab,kf. |
| #41 | Blood Vessel Prosthesis Implantation/ |
| #42 | stents/ or drug-eluting stents/ or self expandable metallic stents/ |
| #43 | (stent* or graft* or endograft* or endoprosthe*).ti,ab,kf. |
| #44 | (sirolimus or nitinol or zilver or IDEV).ti,ab,kf. |
| #45 | Atherectomy/ |
| #46 | atherectom*.ti,ab,kf. |
| #47 | Brachytherapy/ |
| #48 | (brachytherap* or brachy-therap* or curietherap* or curie-therap* or plaque therap* or surface radiotherap*).ti,ab,kf. |
| #49 | (radiotherap* adj1 (implant* or interstitial or intracavit* or intra-cavit*)).ti,ab,kf. |
| #50 | Cryotherapy/ |
| #51 | (cryotherap* or cryoplast* or cryosurg* or cold therap*).ti,ab,kf. |
| #52 | or/34-51 |
| #53 | 11 and ((28 and 52) or 33) |
| #54 | (exp animal experimentation/ or exp models, animal/) not (human experimentation/ or humans/) |
| #55 | exp animals/ not (exp animals/ and humans/) |
| #56 | (rat or rats or mouse or mice or swine or porcine or murine or sheep or lambs or pigs or piglets or rabbit or rabbits or cat or cats or dog or dogs or cattle or bovine or monkey or monkeys or trout or marmoset$1).ti. |
| #57 | review.pt. or (letter or comment*).ti,pt. |
| #58 | (case adj (report? or control*)).ti,hw,pt. |
| #59 | or/54-58 |
| #60 | 53 not 59 |
| #61 | ((coronary or cardiac or cardiovascular or aortic or renal or hepatic) not (peripheral or femoral or femoropopliteal or femor* popliteal or infrapopliteal or infra popliteal or iliac or aortoiliac or aorto iliac or tibial or tibioperoneal or tibio peroneal or tibia fibila? or tibiafibila? or crural or inguinal or inguinal or infrainguinal or pedis or tarsal or planar or malleolar or arcuate or metatarsal)).ti. |
| #62 | 60 not 61 |
| #63 | remove duplicates from 62 |
| #64 | (systematic or structured or evidence or trials or studies).ti. and ((review or overview or look or examination or update* or summary).ti. or review.pt.) |
| #65 | (0266-4623 or 1469-493X or 1366-5278 or 1530-440X or 2046-4053).is. |
| #66 | meta-analysis.pt. or (meta-analys* or meta analys* or metaanalys* or meta synth* or meta-synth* or metasynth*).ti,ab,kf,hw. |
| #67 | ((systematic or meta) adj2 (analys* or review)).ti,kf. or ((systematic* or quantitativ* or methodologic*) adj5 (review* or overview*)).ti,ab,kf,sh. or (quantitativ$ adj5 synthesis$).ti,ab,kf,hw. |
| #68 | (integrative research review* or research integration).tw. or scoping review?.ti,kf. or (review.ti,kf,pt. and (trials as topic or studies as topic).hw.) or (evidence adj3 review*).ti,ab,kf. |
| #69 | review.pt. and ((medline or medlars or embase or pubmed or scisearch or psychinfo or psycinfo or psychlit or psyclit or cinahl or electronic database* or bibliographic database* or computeri#ed database* or online database* or pooling or pooled or mantel haenszel or peto or dersimonian or der simonian or fixed effect or ((hand adj2 search*) or (manual* adj2 search*))).tw,hw. or (retraction of publication or retracted publication).pt.) |
| #70 | or/64-69 |
| #71 | (exp Animals/ or Animal Experimentation/ or exp Models, Animal/) not (exp Human Experimentation/ or Humans/) |
| #72 | (letter or comment).pt. |
| #73 | 70 not (61 or 71 or 72) |
| #74 | 53 and 73 |

**EMBASE search strategy**

| ID | Search |
| --- | --- |
| #1 | randomized controlled trial/ |
| #2 | (RCT or randomi#ed or randomi#ation).ab,ti,kw,hw. |
| #3 | (random* adj3 (administ* or allocat* or assign* or class* or control* or determine* or divide* or distribut* or expose* or fashion* or number* or place* or recruit* or subsitut* or treat*)).ab,kw. |
| #4 | trial.ti. |
| #5 | crossover procedure/ |
| #6 | ((singl* or doubl* or tripl* or trebl*) adj3 (blind* or mask* or dumm*)).ti,ab,kw,hw. |
| #7 | phase 2 clinical trial/ or phase 3 clinical trial/ or phase 4 clinical trial/ |
| #8 | or/1-7 |
| #9 | Cross-sectional study/ not (randomized controlled trial/ or controlled clinical study/ or controlled study/ or randomi?ed controlled.ti,ab. or control group$1.ti,ab. |
| #10 | (((case adj control$) and random$) not randomi?ed controlled).ti,ab. |
| #11 | ((systematic review or meta-analysis) not (trial or study)).ti. |
| #12 | review.pt. not (trial or RCT).ti. |
| #13 | "we searched".ab. and (review.ti. or review.pt.) |
| #14 | "update review".ab. |
| #15 | (databases adj4 searched).ab. |
| #16 | (rat or rats or mouse or mice or swine or porcine or murine or sheep or lambs or pigs or piglets or rabbit or rabbits or cat or cats or dog or dogs or cattle or bovine or monkey or monkeys or trout or marmoset$1).ti. and animal experiment/ |
| #17 | Animal experiment/ not (human experiment/ or human/) |
| #18 | or/9-17 |
| #19 | 8 not 18 |
| #20 | peripheral vascular disease/ |
| #21 | *peripheral occlusive artery disease/ or peripheral occlusive artery disease/dm, pc, th, su |
| #22 | (Atherosclerosis/dm, su, th or Arteriosclerosis/dm, su, th) and (peripher* or limb? or leg?).ti,ab,kw,hw. |
| #23 | *artery occlusion/pc, su, th |
| #24 | in-stent restenosis/pc, su, th |
| #25 | exp *femoral artery/ or exp femoral artery/dm, th, su |
| #26 | ((femoral artery or femoropopliteal or popliteal artery) adj2 (disease or lesion? or stenosis)).hw. |
| #27 | popliteal artery/ |
| #28 | exp *iliac artery/ or exp iliac artery/su |
| #29 | tibial artery/ |
| #30 | (Leg Artery/ or Femoral Artery/ or Popliteal Artery/ or Iliac Artery/ or Tibial Artery/) and (Vascular Patency/ or endovascular surgery/ or vascular surgery/) |
| #31 | leg ischemia/ or leg revascularization/ |
| #32 | (foot/ or lower limb/ or limb salvage/) and (vascularization/ or revascularization/ or ischemia/ or thrombosis/) |
| #33 | ((limb? or leg? or foot or feet or lower extremit* or ((above or below) adj2 (knee? or ankle?))) adj3 (arter* or isch?emi* or lesion? or recanali* or revascular* or segment or thrombo* or surgery or intervention)).ti,ab,kw. |
| #34 | (peripheral adj3 (arter* or vascular*) adj3 (disease or isch?emi* or segment or occlus* or patency or reocclus* or re-occlus* or steno* or restenos* or revascular* or recanali* or obstruct* or lesio* or block* or harden* or stiffen* or obliter* or intervention or surgery)).ti,ab,kw. |
| #35 | (((femoral or femoropopliteal or femor* popliteal or infrapopliteal or infra popliteal or iliac or aortoiliac or aorto iliac or tibial or tibioperoneal or tibio peroneal or tibia fibila? or tibiafibila? or crural or inguinal or inguinal or infrainguinal or pedis or tarsal or planar or malleolar or arcuate or metatarsal) adj2 (arter* or segment)) and (disease or isch?emi* or occlus* or patency or reocclus* or re-occlus* or steno* or restenos* or revascular* or recanali* or obstruct* or lesio* or block* or harden* or stiffen* or obliter* or intervention or surgery)).ti,ab,kw. |
| #36 | ((femoral or femoropopliteal or femor* popliteal or infrapopliteal or infra popliteal or iliac or aortoiliac or aorto iliac or tibial or tibioperoneal or tibio peroneal or tibia fibila? or tibiafibila? or crural or inguinal or inguinal or infrainguinal or pedis or tarsal or planar or malleolar or arcuate or metatarsal) adj3 (isch?emi* or occlus* or patency or reocclus* or re-occlus* or steno* or restenos* or revascular* or recanali* or obstruct* or lesion* or block* or harden* or stiffen* or obliter* or intervention or surgery)).ti,ab,kw. |
| #37 | ((critical or chronic) adj2 limb? adj2 isch?emi*).ti,ab,kw,hw. |
| #38 | critical limb ischemia/ |
| #39 | intermittent claudication/ or claudication/ |
| #40 | claudication.ti,ab,kw. |
| #41 | or/20-40 |
| #42 | ((Admiral or Amphirion or Apex or Chocolate or Cotavance or CVI or Dior or Elutax or EverCross or Freeway or Genie or "IN.PACT" or LegFlow or Lutonix or Moxy or Pacific or Pantera or Range or SeQuent or Stellarex or Zilver) adj2 (balloon? or baloon? or catheter* or stent*)).ti,ab,kw. |
| #43 | angioplasty/ or percutaneous transluminal angioplasty/ or laser angioplasty/ |
| #44 | balloon catheterization/ or balloon occlusion/ or laser-assisted balloon angioplasty/ or balloon catheter/ or brachytherapy balloon catheter/ or percutaneous transluminal angioplasty balloon/ or balloon/ or balloon dilatation/ or occlusion balloon catheter/ or angioplasty catheter/ |
| #45 | drug coated balloon/ or drug eluting balloon/ or lutonix paclitaxel coated balloon/ or paclitaxel coated angioplasty balloon/ |
| #46 | (angioplas* or venoplas* or (percutaneous adj3 (intervention? or procedure? or technique? or treatment?))).ti,ab,kw. |
| #47 | (balloon? or baloon? or dilatation catheter*).ti,ab,kw. |
| #48 | ((stent* or graft* or endograft* or endoprosthe*) adj5 (femoral or femoropopliteal or femor* popliteal or infrapopliteal or infra popliteal or iliac or aortoiliac or aorto iliac or tibial or tibioperoneal or tibio peroneal or tibia fibila? or tibiafibila? or crural or inguinal or inguinal or infrainguinal or pedis or tarsal or planar or malleolar or arcuate or metatarsal or ((above or below) adj2 (knee? or ankle?)))).ti,ab,kw,hw. |
| #49 | ((stent* or graft* or endograft* or endoprosthe*) adj5 peripheral adj5 (arter* or vascular*)).ti,ab,kw. |
| #50 | blood vessel prosthesis/ or artery prosthesis/ |
| #51 | *endovascular surgery/ |
| #52 | ((endovascular* adj3 (intervention? or procedure? or technique? or treatment? or peripheral or percutaneous)) and (femoral or femoropopliteal or femor* popliteal or infrapopliteal or infra popliteal or iliac or aortoiliac or aorto iliac or tibial or tibioperoneal or tibio peroneal or tibia fibila? or tibiafibila? or crural or inguinal or inguinal or infrainguinal or pedis or tarsal or planar or malleolar or arcuate or metatarsal or ((above or below) adj2 (knee? or ankle?)))).ti,ab,kw. |
| #53 | stent/ or metal stent/ or bare metal stent/ or self expanding stent/ or self expandable metallic stent/ or vascular stent/ or peripheral artery stent/ or femoral artery stent/ or superficial femoral artery stent/ or popliteal artery stent/ or femoropopliteal stenting/ or iliac artery stent/ or drug eluting stent/ or nitinol stent/ or drug eluting nitinol stent/ or self expanding nitinol stent/ |
| #54 | ((sirolimus or nitinol or zilver or IDEV or drug eluting) adj3 stent*).ti,ab,kw. |
| #55 | atherectomy/ or directional atherectomy/ or rotational atherectomy/ |
| #56 | atherectom*.ti,ab,kw. |
| #57 | exp brachytherapy/ |
| #58 | (brachytherap* or brachy-therap* or curietherap* or curie-therap* or plaque therap* or surface radiotherap*).ti,ab,kw. |
| #59 | (radiotherap* adj1 (implant* or interstitial or intracavit* or intra-cavit*)).ti,ab,kw. |
| #60 | exp cryotherapy/ |
| #61 | (cryotherap* or cryoplast* or cryosurg*).ti,ab,kw. |
| #62 | or/43-61 |
| #63 | ((coronary or cardiac or cardiovascular or aortic or renal or hepatic or thoracic or upper extremit* or bone marrow) not (peripheral or femoral or femoropopliteal or femor* popliteal or infrapopliteal or infra popliteal or iliac or aortoiliac or "aorto iliac" or tibial or tibioperoneal or tibio peroneal or tibia fibila* or tibiafibila* or crural or inguinal or inguinal or infrainguinal or pedis or tarsal or planar or malleolar or arcuate or metatarsal or lower extremit*)).ti. |
| #64 | (19 and 42) or (19 and 41 and 62) |
| #65 | 64 not 63 |

**Cochrane search strategy**

| ID | Search |
| --- | --- |
| #1 | MeSH descriptor: [Peripheral Vascular Diseases] explode all trees |
| #2 | MeSH descriptor: [Arterial Occlusive Diseases] this term only |
| #3 | MeSH descriptor: [Arteriosclerosis] this term only |
| #4 | MeSH descriptor: [Arteriosclerosis Obliterans] this term only |
| #5 | MeSH descriptor: [Intermittent Claudication] this term only |
| #6 | MeSH descriptor: [Atherosclerosis] this term only |
| #7 | MeSH descriptor: [Peripheral Arterial Disease] this term only |
| #8 | MeSH descriptor: [Femoral Artery] this term only |
| #9 | MeSH descriptor: [Popliteal Artery] this term only |
| #10 | MeSH descriptor: [Iliac Artery] this term only |
| #11 | MeSH descriptor: [Tibial Arteries] this term only |
| #12 | (surgery or therapy or pathology):kw |
| #13 | ((#8 or #9 or #10 or #11) and #12) |
| #14 | MeSH descriptor: [Vascular Patency] this term only |
| #15 | MeSH descriptor: [Vascular Surgical Procedures] explode all trees |
| #16 | (#8 or #9 or #10 or #11) and (#14 or #15) |
| #17 | MeSH descriptor: [Limb Salvage] this term only |
| #18 | MeSH descriptor: [Leg] this term only and with qualifier(s): [Blood supply - BS] |
| #19 | MeSH descriptor: [Foot] explode all trees and with qualifier(s): [Blood supply - BS] |
| #20 | MeSH descriptor: [Lower Extremity] explode all trees and with qualifier(s): [Blood supply - BS] |
| #21 | MeSH descriptor: [Ischemia] this term only |
| #22 | "revascularization":ti,ab,kw (Word variations have been searched) |
| #23 | MeSH descriptor: [Embolism and Thrombosis] explode all trees |
| #24 | (#17 or #18 or #19 or #20) and (#21 or 23 or #23) |
| #25 | ((limb* or leg* or foot or feet or "lower extremit*" or ((above or below) near/2 (knee* or ankle*))) near/3 (arter* or ischemi* or ischaemi* or lesion* or recanali* or revascular* or segment or thrombo* or surgery or surgical or intervention)):ti,ab,kw |
| #26 | ((peripheral near (arter* or vascular*)) near (disease or ischemi* or ischaemi* or segment or occlus* or patency or reocclus* or re-occlus* or steno* or restenos* or revascular* or recanali* or obstruct* or lesio* or block* or harden* or stiffen* or obliter* or intervention or surgery or surgical)):ti,ab,kw |
| #27 | (((femoral or femoropopliteal or "femor* popliteal" or infrapopliteal or "infra popliteal" or iliac or aortoiliac or aorto iliac or tibial or tibioperoneal or "tibio peroneal" or "tibia fibila*" or tibiafibila* or crural or inguinal or inguinal or infrainguinal or pedis or tarsal or planar or malleolar or arcuate or metatarsal) near/2 (arter* or segment)) and (disease or ischemi* or ischaemi* or occlus* or patency or reocclus* or re-occlus* or steno* or restenos* or revascular* or recanali* or obstruct* or lesio* or block* or harden* or stiffen* or obliter* or intervention or surgery or surgical)):ti,ab,kw |
| #28 | ((femoral or femoropopliteal or "femor* popliteal" or infrapopliteal or "infra popliteal" or iliac or aortoiliac or "aorto iliac" or tibial or tibioperoneal or "tibio peroneal" or "tibia fibila*" or tibiafibila* or crural or inguinal or inguinal or infrainguinal or pedis or tarsal or planar or malleolar or arcuate or metatarsal) near/3 (ischemi* or ischaemi* or occlus* or patency or reocclus* or re-occlus* or steno* or restenos* or revascular* or recanali* or obstruct* or lesion* or block* or harden* or stiffen* or obliter* or intervention or surgery or surgical)):ti,ab,kw |
| #29 | (((critical or chronic) near limb*) near (ischemi* or ischaemi*)):ti,ab,kw |
| #30 | claudication:ti,ab,kw |
| #31 | #1 or #2 or #3 or #4 or #5 or #6 or #7 or #13 or #16 or #24 or #25 or #26 or #27 or #28 or #29 or #30 |
| #32 | ((femoral or femoropopliteal or "femor* popliteal" or infrapopliteal or "infra popliteal" or iliac or aortoiliac or "aorto iliac" or tibial or tibioperoneal or "tibio peroneal" or "tibia fibila*" or tibiafibila* or crural or inguinal or inguinal or infrainguinal or pedis or tarsal or planar or malleolar or arcuate or metatarsal or ((above or below) near/2 (knee* or ankle*))) near/2 (balloon* or baloon* or angioplas* or stent*)):ti,ab,kw |
| #33 | ((Admiral or Amphirion or Apex or Chocolate or Cotavance or CVI or Dior or Elutax or EverCross or Freeway or Genie or "IN.PACT" or LegFlow or Lutonix or Moxy or Pacific or Pantera or Range or SeQuent or Stellarex or Zilver) near/2 (balloon* or baloon* or catheter* or stent*)):ti,ab,kw |
| #34 | ((Admiral or Amphirion or Apex or Chocolate or Cotavance or CVI or Dior or Elutax or EverCross or Freeway or Genie or "IN.PACT" or LegFlow or Lutonix or Moxy or Pacific or Pantera or Range or SeQuent or Stellarex or Zilver) near (femoral or femoropopliteal or "femor* popliteal" or infrapopliteal or "infra popliteal" or iliac or aortoiliac or "aorto iliac" or tibial or tibioperoneal or "tibio peroneal" or "tibia fibila*" or tibiafibila* or crural or inguinal or inguinal or infrainguinal or pedis or tarsal or planar or malleolar or arcuate or metatarsal or ((above or below) near/2 (knee* or ankle*)))):ti,ab,kw |
| #35 | ((peripheral or percutaneous) and endovascular):ti |
| #36 | #32 or #33 or #34 or #35 *[Intervention - Precise]* |
| #37 | MeSH descriptor: [Angioplasty] explode all trees |
| #38 | (angioplas* or venoplas* or (percutaneous near/3 (intervention* or procedure* or technique* or treatment*))):ti,ab,kw |
| #39 | (balloon* or baloon* or "dilatation catheter*"):ti,ab,kw |
| #40 | ((PTA or DEB or DCB or PEB or PCB or paclitaxel or PTX or sirolimus or nitinol or zilver or IDEV) near (femoral or femoropopliteal or "femor* popliteal" or infrapopliteal or "infra popliteal" or iliac or aortoiliac or aorto iliac or tibial or tibioperoneal or "tibio peroneal" or "tibia fibila*" or tibiafibila* or crural or inguinal or inguinal or infrainguinal or pedis or tarsal or planar or malleolar or arcuate or metatarsal or ((above or below) near/2 (knee* or ankle*)))):ti,ab,kw |
| #41 | ((stent* or graft* or endograft* or endoprosthe*) near/2 (femoral or femoropopliteal or "femor* popliteal" or infrapopliteal or "infra popliteal" or iliac or aortoiliac or aorto iliac or tibial or tibioperoneal or "tibio peroneal" or "tibia fibila*" or tibiafibila* or crural or inguinal or inguinal or infrainguinal or pedis or tarsal or planar or malleolar or arcuate or metatarsal or ((above or below) near/2 (knee* or ankle*)))):ti,ab,kw |
| #42 | (((stent* or graft* or endograft* or endoprosthe*) near peripheral) near (arter* or vascular*)):ti,ab,kw |
| #43 | ((stent* or graft* or endograft* or endoprosthe*) near (limb* or leg* or foot or feet or "lower extremit*")):ti,ab,kw |
| #44 | MeSH descriptor: [Endovascular Procedures] this term only |
| #45 | endovascular.ti. or (endovascular* next (intervention* or procedure* or technique* or treatment*)):ti,ab,kw |
| #46 | MeSH descriptor: [Blood Vessel Prosthesis Implantation] this term only |
| #47 | MeSH descriptor: [Stents] explode all trees |
| #48 | ((PTA or DEB or DCB or PEB or PCB or paclitaxel or PTX or sirolimus or nitinol or zilver or IDEV or "drug eluting") near/3 stent*):ti,ab,kw or (stent* or graft* or endograft* or endoprosthe*):ti |
| #49 | atherectom*:ti,ab,kw |
| #50 | MeSH descriptor: [Brachytherapy] this term only |
| #51 | (brachytherap* or brachy-therap* or curietherap* or curie-therap* or "plaque therap*" or "surface radiotherap*"):ti,ab,kw |
| #52 | (radiotherap* near/2 (implant* or interstitial or intracavit* or intra-cavit*)):ti,ab,kw |
| #53 | MeSH descriptor: [Cryotherapy] this term only |
| #54 | (cryotherap* or cryoplast* or cryosurg*):ti,ab,kw |
| #55 | #37 or #38 or #39 or #44 or #45 or #46 or #47 or #48 or #49 or #50 or #51 or #52 or #53 or #54 *[Intervention - Broad]* |
| #56 | **#36 or (#31 and #55) or #40 or #41 or #42 or #43** |
| #57 | HS-PVD or (SR-PVD and (conference or symposi* or seminar* or proceeding* or workshop* or colloqui* or session or assembl* or meeting* or congress* or summit* or forum or societ* or college)) |
| #58 | #57 and (#26 or #27 or #28 or #29 or #30 or #38 or #39 or #45 or #48 or #49 or #51) |
| #59 | **#56 or #58** |
| #60 | *((coronary or cardiac or cardiovascular or aortic or renal or hepatic or thoracic or "upper extremit*" or "bone marrow") not (peripheral or femoral or femoropopliteal or "femor* popliteal" or infrapopliteal or "infra popliteal" or iliac or aortoiliac or "aorto iliac" or tibial or tibioperoneal or "tibio peroneal" or "tibia fibila*" or tibiafibila* or crural or inguinal or inguinal or infrainguinal or pedis or tarsal or planar or malleolar or arcuate or metatarsal or "lower extremit*")):ti* |
| #61 | **#59 not #60** |
